# The challenges facing programs for the prevention and control of non-communicable diseases in Iran: a qualitative study of senior managers' viewpoints

**DOI:** 10.1186/s12913-022-08778-6

**Published:** 2022-11-15

**Authors:** Yegane Partovi, Mostafa Farahbakhsh, Jafar Sadegh Tabrizi, Kamal Gholipour, Ahmad Koosha, Jabreil Sharbafi, Andrew Wilson

**Affiliations:** 1 Department of Health Policy and Health Services Management, School of Management and Medical Informatics, Tabriz University of Medical Sciences, Tabriz, Iran; 2 Research Center of Psychiatry and Behavioral Sciences, Tabriz University of Medical Sciences, Tabriz, Iran; 3 Health Services Management Research Centre, School of Health Management and Medical Information, Tabriz University of Medical Sciences, Tabriz, Iran; 4 Health Services Management Research Center, School of Management and Medical Informatics, Tabriz University of Medical Sciences, Tabriz, Iran; 5 National Public Health Management Center, School of Public Health, Tabriz University of Medical Sciences, Tabriz, Iran; 6 East Azerbaijan Provincial Health Centre, NCD Department, Tabriz, Iran; 7 Menzies Centre for Health Policy, School of Public Health, the University of Sydney, Sydney, Australia

**Keywords:** Qualitative Study, Non-Communicable Diseases, Prevention and Control

## Abstract

**Background:**

Despite significant achievements in the prevention and control of NCDs in Iran, these conditions are still the biggest challenges to Iran's healthcare system and are estimated to account for 78.1 percent of all deaths. Therefore, this study aimed to reflect on the potential challenges standing in the way to implement the relevant policies, empower the dimensions of governance, and react in an effective and timely manner by Iran's healthcare system to NCDs.

**Methods:**

This study was conducted with a qualitative approach using the conventional content analysis method. A total of 46 senior managers involved in the prevention and control of NCDs at the medical sciences universities across Iran were recruited through the purposive sampling method and were interviewed via semi-structured interviews. Graneheim and Lundman's approach was utilized to analyze the data.

**Results:**

According to the analysis of the senior managers' viewpoints, current challenges to implement the program for the prevention and control of NCDs in Iran could be placed into six main categories, including financing, human resources, infrastructure and inputs, legal, executive, administrative, as well as inter-sectoral collaboration, and management and policy-making challenges with their own sub-categories.

**Conclusion:**

The results revealed that financing was the biggest challenge to successfully implementing the program for the prevention and control of NCDs in Iran. However, strengthening Iran's healthcare system in the field of the prevention of NCDs demanded more innovative measures and strategies, such as the empowerment of human resources, the effective use of intra- and inter-sectoral collaboration, and non-governmental organizations and charities, along with the exploitation of evidence-based studies during policy-making and decision-making processes, with no need for financial resources.

## Introduction

In 2019, 74% of all deaths and 63.8% of disability-adjusted life years (DALY) had occurred due to NCDs [1]. According to the World Health Organization (WHO), such diseases take the lives of 15 million individuals aged 30-69 each year, with more than 80% of premature death happening in developing and low-income countries [2]. As a middle-income nation, Iran, recorded 83.5% of mortalities and 78.1% of the total disease burden in 2019 that were related to NCDs [1, 3, 4]. Accordingly, coronary artery disease, low back pain, road traffic crashes, major depressive disorder, and stroke had the highest burdens in Iran in 2010. This trend was not similar to the pattern of NCDs in developing countries but that in developed nations [5, 6]. Given the rising prevalence rates of such diseases worldwide and as a priority for sustainable development in 2018-2030 (according to the Non-Communicable Diseases Roadmap (3rd Meeting)), all countries were required to reduce NCDs caused premature death rates by up to 25% until 2025 based on nine voluntary global targets and recommended interventions [5, 7–9].

To successfully realize such targets in Iran, several measures, including the Development of National Documents on the Prevention and Control of Non-Communicable Diseases, the establishment of the National Committee for the Prevention and Control of Non-Communicable Diseases, the formulation of the National Action Plan for NCDs 2015-2020, and the implementation of the WHO Essential Intervention Package (IraPEN) in the field of primary healthcare (PHC) have been considered. Recent research on the implementation of the WHO policies in 151 countries also shows that Iran and Costa Rica have obtained the highest scores (86.1%) with a mean value of 49.3 [10]. Similarly, in early 2017, the WHO officially declared Iran as a fast-track country in preventing and controlling NCDs. In October 2017, Iran was further applauded in the WHO Non-Communicable Diseases Conference in Uruguay for its success in fighting against NCDs [11]. Likewise, in September 2018, at the UN Summit in New York in the United States, Iran was acknowledged for its outstanding contribution to preventing and controlling chronic diseases and mental illnesses related to the Sustainable Development Goals [11].

Nevertheless, studies demonstrate that most countries have faced challenges in implementing the WHO policies and recommendations so far. For example, the results of one study in seven Asian countries in 2018 revealed that the most important obstacles to the implementation of the WHO policies were insufficient budgets, limited organizational capacities, poor sectoral coordination, and lack of formal monitoring and evaluation activities [12]. Despite many achievements in the prevention and control of NCDs in Iran, such conditions are still the most significant challenges that Iran's healthcare system faces. Accordingly, problems facing the healthcare services' rationing and referral systems, lack of finance, defects in health information management (HIM), and the use of private sector capacity and effective inter-sectoral collaboration are among the dozens of other challenges hindering the process of combating NCDs. To identify and eliminate such challenges interfering with the implementation of the relevant policies, empowering the dimensions of governance, and effective and timely responsiveness by Iran's healthcare system to NCDs, this study was conducted based on a qualitative approach and conventional content analysis method. The study design of most previous NCDs-related studies have been quantitative and review, and most had investigated the analysis of policies to risk factors of NCDs in Iran, but fewer cases had examined challenges facing the programs for the prevention and control of non-communicable diseases [4, 13–19]. We used qualitative research to evaluate and report the complexity and the hidden aspects of the challenges, which this was not feasible with quantitative approaches [20]. More specifically, in this study, the viewpoints of managers, policy-makers, and experts involved in programs for the prevention and control of NCDs were evaluated, and gave us deep and comprehensive information about the challenges facing the programs for the prevention and control of non-communicable diseases, thereby increasing our understanding of these challenges.

## Method

This study was a qualitative study and used the conventional content analysis method. We used COREQ (Consolidated criteria for Reporting Qualitative research) Checklist to direct our study [21].

### Participants and sampling

The study target population included all provincial health managers of the program for prevention and control of NCDs at the medical sciences universities across Iran. These individuals are responsible for planning and monitoring the provincial Action Plan for NCDs at the provincial level. The list of provincial health managers was received from the National Committee for the Prevention and Control of Non-Communicable Diseases. Then, using the purposive sampling method, we recruited 46 provincial health managers who were undergone semi-structured interviews. Data were collected from February 2020 to April 2021. The inclusion criteria consisted of having at least three years of management experience or executive activities in the field of PHC and NCDs and having the qualifications and willingness to attend the interviews. Exclusion criteria included the unwillingness and lack of interest in participating in the study.

### Data collection

A total of 46 interviews were conducted with managers. The interviews were conducted in Farsi (Persian), and each interview lasted 30 minutes on average. The interviews were conducted in the interviewees' workplaces (36 interviews) or via phone calls (10 interviews ) with some senior managers who were in other far provinces. Before performing the interview, a signed informed consent form was obtained from all the participants after they were explained about the confidentiality of the data. In some cases, some managers were hesitant to participate due to political considerations, day-to-day responsibilities, and institutional positions. The research team informed respondents about the study's importance and obtained the consent of the participants with the support of the National Committee for the Prevention and Control of Non-Communicable Diseases and assured them that this research would not publish their names. The interview guideline was further designed after reviewing the literature and conducting five in-depth pilot interviews. The interviewer reviewed the study's aims with each interviewee and then asked questions step by step while encouraging the interviewee to discuss every question freely. All interviews were recorded with participants consent. More details about questions are provided in Table 1. A research assisstant took note during each interview and assisted the interviewer. The interviews were also halted when the researchers determined that more interviews would not provide them with new information [22].

**Table 1:** Some general questions

Raw	Questions
1	How is the health system performance of chronic disease programs? On the whole, how would you describe the current status of chronic disease programs?
2	What factors hinder the performance of the health system in chronic disease programs? Please Explain
	What factors do you think support the health system's performance in chronic disease programs? Please Explain (Organization; precise planning; good budgeting; motivation., etc.)
3	What are the potential challenges in chronic disease programs? Can you provide an example? (lack of resources; control over finances and planning; staff turnover., etc.)
4	Which of mentioned challenges do you think is most important? Please Explain.

### Data analysis

To analyze the data, we followed Graneheim and Lundman's approach [23]. Preliminary analysis and coding of the data from each interview were performed before starting the next interview. Immediately after ending the interviews, the recorded contents were transcribed word by wordfrom the audio recorder. Then, the transcripts were matched with the notes taken during the interview sessions, and finally, a single text was reached. During the data preparation stage, the participants' viewpoints were subsequently read several times to better understand the texts and immerse in them. At the defining meaning units stage, the meaning units were extracted and categorized as condensed units. The condensed meaning units were abstracted and labeled with a code. The codes were also grouped into different categories based on their differences or similarities, which was performed independently by two researchers as the research team members. Ultimately, the data were adjusted through discussions and the resolutions of some disagreements during a meeting.

#### The rigor of the study

To augment the consistency and accuracy of the study results, the four criteria of credibility, confirmability, dependability, and transferability proposed by Guba and Lincoln (1985) were applied [24]. For credibility and confirmability purposes, we employed immersion and peer checking and expert opinions and participant reviews. In this way, after completing the interview sessions and recapping the viewpoints of the study participants, a summary of the statements from the notes taken during the interviews was told to participants to correct or eliminate the wrong and vague cases. The transcribed interviews were also provided to the participants to confirm and correct possible discrepancies. Considering dependability, two individuals were further recruited for coding. Moreover, expert opinions and the purposive sampling method were used to meet transferability [25].

## Results

As shown in Table 2, 54% of the participants were female, and over 60% of the participants had 21 to 30 years of work experience. In terms of the levels of education, above 67% of the participants had a Ph.D. degree or medical doctorates and higher degrees, and the majority (exceeding 67%) of them were physicians (For more information, see Table 2).

**Table 2:** The study participants' demographic characteristics information

Variables		Frequency	Percentage
Gender	Male	21	45.64
	Female	25	54.3
Job experience	10-5	2	4.34
	20-11	16	34.78
	30-21	28	60.86
Degree of education	Graduate	5	10.86
	Post graduate	10	21.73
	MD/Ph.D.	31	67.39
Field of education	Physician	31	67.39
	Public health, epidemiologist	9	19.56
	Health care management	4	8.69
	Nurse, midwife	2	4.34
Total		46	100

The analysis of the viewpoints of the senior managers involved in the prevention and control of NCDs indicated that the current challenges interfering with the implementation of the programs for the prevention and control of NCDs in Iran were placed into six main categories, including financing, human resources, infrastructure and inputs, legal, executive, administrative, inter-sectoral collaboration, and management and policy-making with their own sub-categories as follows. The summary of the main categories and their sub-categories can be retrieved from Table 3.

**Table 3:** A summary of categories and sub-categories based on the senior managers' viewpoints toward the program for the prevention and control of NCDs

Categories	Sub-categories
Financing	Lack of sustainable finance
	Insufficient allocations of specialized budgets for NCDs
Human Resource (HR)	Improper distribution and shortage of HR
	HR's insufficient knowledge and skills
	Lack of motivation in HR
Infrastructure and inputs	Inconsistency between programs and infrastructure and the capacity of universities of medical sciences
	Shortcomings in HIM system
	Weaknesses in evaluations and lack of a comprehensive evaluation system
Legal, executive, and administrative	Non-allocation of organizational positions and administrative charts for personnel involved in programs
	Inadequacy of salaries and benefits with the volume of program activities
	Lack of transparency in job descriptions at different units and deputies concerning programs
Inter-Sectoral Collaboration	Lack of the Understanding of Program Policies and Objectives by Units, Organizations, and Other Bodies
	Weaknesses in the Skills Related to Seeking Support of Experts and Managers in terms of Attracting Intersectoral Collaboration
	Lack of the Transparency in the Role of NGOs, Charities, and Volunteer Groups
Management and policy-making	Insufficient Support of National and Regional Policy-Makers and Lack of Understanding of Policies and Goals related to NCDs by Senior Managers
	Individual-Dependent Programs and Goals
	Weaknesses in Evidence-Based Decision-Making and Policy-Making System

### Financing

#### Lack of sustainable finance

Among the most important challenges raised by the participants were financing and the sustainability and continuity of financial resources. From their viewpoints, finance resources were one of the practical issues in implementing and fulfilling the programs for the prevention and control of NCDs. In this regard, one of the senior managers stated that:

"*In my idea, financing in a continuous and sustainable manner is assumed to be one of the critical factors to the success and implementation of the program to prevent and control non-communicable diseases. Unfortunately, in our country, there are no fixed and coherent financial resources dedicated to such healthcare programs, specially for the management of non-communicable diseases*." (Participant NO.3)

#### Insufficient allocations of specialized budgets for NCDs

One of the challenges addressed by the study participants in the financing domain was the allocation of specialized budgets for the prevention and control of NCDs. In this respect, a senior manager stated that:

"*There is insufficiency and shortages per capita and even non-compliance with the current needs in Iran. I think the allocation of credits in the Ministry of Health and Medical Education does not happen properly, and they are quite unfair. For example, the National Diabetes Prevention Program launched successfully in the early days, but after a while, due to lack of financial resources, it faced many problems as a result of which the program did not achieve satisfactory outcomes.*" ( Participant NO.6)

### Human Resource (HR)

#### Improper distribution and shortage of HR

HR was introduced as the input for the implementation of the program for the prevention and control of NCDs, and the study participants discussed its shortages and improper distribution affecting the implementation of the associated program. For example, one of the participants reiterated that:

*"Day after day, new programs and activities are added, but the existing workforce is not able to meet them. I mean, the number of such programs is large enough, but the number of the personnel required is low, and the workload is ever-increasing*." (Participant NO.10)

Another senior manager also added that:

"*There has been a mismatch between the supply and demand of workforce in Iran's healthcare system from the very beginning, and this challenge is also evident in the program for the prevention and control of non-communicable diseases. I think spatial and temporal imbalances along with the improper distribution of human resources have been among the problems facing our healthcare system, and now they are interrupting the programs dedicated to the management of non-communicable diseases*." ( Participant NO.14)

#### HR's insufficient knowledge and skills

Related experties and professional workforce to implement the program for the prevention and control of NCDs were also addressed by the senior managers as one of the challenges in the field of HR. In this regard, one of the participants said that:

"*There are no experts to implement such programs. Our personnel does not have enough skills to manage, analyze, and use information related to their fields. The same retired workforce has been rehired in many units, and they continue to work. Considering such shortcomings, we need to stop expecting more*." ( Participant NO.7)

#### Lack of motivation in HR

Among the other HR challenges raised by the participants was the lack of motivation among personnel and reduced job satisfaction. For example, one of the participants stated that:

*"In recent years, a wide variety of programs have been delivered to the medical sciences universities, which take a significant amount of time from experts. Apart from the genetic program, which was very understandable, the rest was vague in many cases and had no outputs at the end of the year, which could discourage all and reduce their levels of job satisfaction." (* Participant NO.*8)*

Moreover, another participant believed that:

*"The level of services required in implementing the program for the prevention and control of non-communicable diseases is higher than the capacities of the workforce. Their personnel's economic problems have further affected their performance and decreased their levels of job satisfaction.*" ( Participant NO.20)

### Infrastructure and inputs

#### Inconsistency between programs and infrastructure and capacity of medical sciences universities

Among the problems addressed in the senior managers' viewpoints in the field of infrastructure and inputs was the inconsistency between programs and infrastructure and capacity of medical sciences universities, which included limited access to physicians, paraclinical services, lack of the full insurance coverage of services and screenings, and defects in healthcare services rationing and referral systems.

***"****There is still a lack of access to physicians and medications, especially for diabetes and hypertension. Also, there is the lack of diagnostic services in many areas in Iran." ( Participant NO.15)*

*"Insurance companies do not cover screening services. Unfortunately, many services are still not covered by insurance. There is also no understanding of the importance of making investments in such services by insurance companies, which can reduce future treatment costs." ( Participant NO.35)*

*"There is no consistency between the structure of Iran's healthcare system and the requirements of the program for the prevention and control of non-communicable diseases. There is also no perfect basic infrastructure such as healthcare services rationing and referral systems in our country. I think our healthcare system has not been developed for the prevention and control of such diseases at all, and this is a big problem." ( Participant NO.27)*

#### Weaknesses in evaluations and lack of a comprehensive evaluation system

In this regard, one of the participants stated that:

*"There is no coherent monitoring and supervision program specified for health risk factors at the level of community. I think they aimed to support the implementation of Article 37 of the given program, but it has been performed incompletely." (* Participant NO.27)

#### Shortcomings in HIM System

One of the main challenges raised by the participants was the problems with the HIM system in all aspects, including data collection, processing, exploitation, credibility, and reporting. For example, one of the senior managers stated that:

*"In my idea, there are no specific standards in terms of the circulation of information, data collection, and even the way to use and analyze them."* (Participant NO.45)

*"Even the SIB as an integrated health system is not appropriate for reporting. As well, the statistics and figures obtained from this system do not have the basic accuracy and credibility*." ( Participant NO.9)

### Legal, executive, and administrative challenges

#### Non-allocation of organizational positions and administrative charts for personnel involved in the program

One of the frequent challenges addressed by the senior managers was the non-allocation of organizational positions and administrative charts for the personnel affiliated with the program for the prevention and control of NCDs. For example, one of the participants added that:

"*The lack of organizational positions related to the program makes the healthcare workers unmotivated. In addition to allocating budgets and credits for non-communicable diseases, an organizational-administrative chart is also required*." ( Participant NO.33)

#### Inadequacy of salaries and benefits with the volume of program activities

To describe this issue, one of the participants said that:

"*The organizational chart has faced many problems. Moreover, the large volume of activities related to each program, and as a result, the received salaries and benefits compared with the volume of work performed are not consistent, and they really discourage the personnel*." ( Participant NO.40)

#### Lack of transparency in job descriptions at different units and deputies concerning the program

Regarding this issue, one of the senior managers stated that:

"*The notified programs are not transparent enough, and the job descriptions at different units and deputies are not further specified. It is unclear how the activities will be done, by whom, at what levels, and with which funding. There is a complete lack of transparency*." ( Participant NO.26)

### Inter-sectoral collaboration

#### Lack of understanding of program policies and objectives by units, organizations, and other bodies

Among the main concerns raised in this regard was the cross-sectoral collaboration to reduce the risk factors and the burden of NCDs. In this line, the most critical issue addressed by the participants was the lack of understanding the importance of the policies developed for the program for the prevention and control of NCDs by other cross-sectoral organizations. In this respect, one of the senior managers said that:

"*Unfortunately, the expectations of the Ministry of Health and Medical Education from other sectors are not clear enough, especially regarding the most important causes of mortality and morbidity*." ( Participant NO.11)

#### Weaknesses in the skills related to seeking support of experts and managers in terms of attracting intersectoral collaboration

The participants referred to the issue of seeking support from and involving other units as a key factor in dealing with NCDs. For example, one of the senior managers said that:

"*The success of the maternal, under-one, and under-five mortality rates, which occurred in the 1980s, owes much to the efforts of other sectors, including the provision of drinking water, the improvement of roads in rural areas, literacy, and the like. It turns out that cross-sectoral organizations have enough capacity to provide services and facilitate the Ministry of Health and Medical Education, provided that the given ministry, senior managers, and experts ask them to get involved and even identify their duties and responsibilities to achieve the goals of the program for the management of non-communicable diseases*." (Participant NO.19)

#### Lack of transparency in the role of NGOs, charities, and volunteer groups

Another issue mentioned by the study participants was the use of the capacity of NGOs and charities to combat NCDs. Accordingly, one of the senior managers believed that:

"*I think charities can provide services to target groups in all three domains of prevention, treatment, and rehabilitation. Unfortunately, the public sector and the unsustainable budgets cannot meet the program's needs for the prevention and control of non-communicable diseases. In my idea, NGOs and charities have their own hidden capacities that can be used as the auxiliaries of the Ministry of Health and Medical Education in dealing with non-communicable diseases. Still, in practice, this is not the case*." ( Participant NO.31)

### Management and policy-making

#### Insufficient support of national and regional policy-makers and lack of understanding of policies and goals related to NCDs by senior managers

The issue of the support and commitment by policy-makers and senior managers in order to understand the goals of policies and help implement them was another important challenge raised by the participants. In this regard, one of the senior managers believed that:

"*The university heads need to be sensitive to the issue of combating non-communicable diseases. There is a tendency to reluctance and underestimation of this program among administrators. As long as university heads and managers have no trust in such programs, all these actions are a waste of time." (* Participant NO.*42)*

Moreover, another participant added that:

"*Unfortunately, the program for the prevention and control of non-communicable diseases is merely spoken but not practiced. Of note, the attitudes of policy-makers and their support and understanding can play a decisive role in the long-term effects of such programs*." (Participant NO.13)

#### Individual-dependent programs and goals

One of the challenges addressed by the study participants was the instability of senior management levels and the rapid relocations of managers in a way that had made programs and goals dependent on the existence of those people. For example, one of the participants stated: "*Immediate changes of the managers and the resultant instability have affected the program's continuation. If there is a fixed program with a stable method, the change of the management will not harm the persistence of the program process.*" (Participant NO.3)

As well, one other participant reiterated that:

"*When the period of management is short, the programs do not have the necessary stability and expertise*." (Participant NO.34)

#### Weaknesses in evidence-based decision-making and policy-making system

In this regard, one of the senior managers stated that:

"*Studies in various areas of healthcare and reflections on knowledge gaps have been thus far very limited, and, oddly, we do not make use of such studies and evidence.*" *(*Participant NO.*27)*

Moreover, another participant believed that:

*We have conducted much research in this respect, but many of our policy-makers are unaware of them because we do not have a comprehensive system for recording these studies and evidence*." (Participant NO.39)

## Discussion

This study aimed to explore the challenges interfering with the implementation of the program for the prevention and control of NCDs in Iran from the viewpoints of senior managers. The challenges from the viewpoints of senior managers who are involved in the healthcare system were placed into six main categories: financing, human resources, infrastructure and inputs, legal, executive, administrative, inter-sectoral collaboration, and management and policy-making challenges with their own sub-categories. The present study results revealed that one of the important challenges in achieving the program's goals for the prevention and control of NCDs and obtaining effective results was financing, which included the lack of sustainability of financial resources and insufficient allocations of specialized budgets for NCDs. Of note, financing was not merely possible through government resources, and at a time that was not long from now, the planned programs would be suspended due to the depletion of the necessary credits and budgets, especially in combating NCDs. Obviously, one of the reasons for the lack of financial resources and instability could be the lack of transparency in financing. In this sense, the lack of concentrated financial resources and health expenditures in a specific position, the dispersion of available resources, variation in costs, and uncertainties about the levels of participation by the public and private sectors, households, and charities could further undermine the sustainability of financial resources [26, 27]. In line with our findings, funding stability problems on the program for the prevention and control of NCDs is a common challenge described in previous studies [28–32]. The insufficient allocations of budgets for NCDs were novel findings in the study that have not been studied in Iran [3, 4, 6, 11, 33, 34]. Major problems such as low per capita allocated to healthcare services, unfair distribution of credits at the national level, and the priority of treatment budgets over the budgets on prevention and PHC (because the latter lack in rapid financial returns) had always been addressed among the permanent challenges [35]. However, in order to implement general health policies for a low per capita allocated to healthcare services, the share of health in the gross domestic product had increased to 7.6% as well as 22.6% of the general government budget in 2015, which was higher in comparison to other neighbor countries with the same ranking [36]. However, the problem was that among the deputies and at the smaller scale, i.e., healthcare programs, the rational distribution of budgets had not been performed with the allocation efficiency, and the credits were not commensurate with the burden of diseases, their prevalence rates, and risk factors.

The findings of numerous studies have further revealed that this issue was not restricted to Iran, and the distribution of budgets for the prevention and control of NCDs had been even disregarded in the international scene. For example, according to the global statistics released between 2000 and 2015, only 1.3% of development aids had been allocated to NCDs despite the fact that such conditions accounted for 50% of the total disease burden in low- and middle-income nations [12]. The study of the implementation of the WHO interventions in seven Asian countries had also indicated that one of the most important obstacles to the implementation of the associated policies was insufficient budgets. Thus, the only way out of financial constraints and instability was to create innovative resources, including earmarked taxes, taxes on goods harmful to health, financial transparency in order to generate accurate information about revenue and spending, equitable access to this information for all stakeholders, and ultimately preventing these resources from being consumed on expenditures other than those in healthcare programs [12].

Another category obtained from participants' views was the human resources, which consisted of improper distribution and shortage, insufficient knowledge and skills, and the lack of motivation of human resource. Unfortunately, in Iran, the education system is not consistent with the needs of the healthcare sector, especially for combating NCDs. Despite the presence of various educational platforms and facilities, including universities and research institutes of medical sciences, specialized scientific associations, and various journals and databases, the scientific strength of healthcare workers has been evaluated as weak. More specifically, in rural areas, the priority in recruiting PHC workers is with local individuals and applicants. This strategy results in hiring workforces with low educational levels, insufficient training, and low skills who can not meet the current needs of different populations [37, 38]. On the other hand, rehiring retired workforce and those with very long service records was a new finding in the study. Naturally, elderly and retired employees do not have the necessary efficiency because of out-of-date knowledge and training, which are not consistent with the actual conditions of society. They would hinder the dynamism and progress of such programs, questioning the benefits of multidisciplinary healthcare teams [39]. Basic measures such as revising educational curricula with the participation of the deputy of health at the ministry and university levels, the use of multidisciplinary technical teams, giving sufficient authority to healthcare managers to select the required personnel based on meritocracy, and preventing the interferences of other units ,and offering job enrichment programs are thus required [39, 40]. Previous studies have further shown that inefficiencies in knowlegde to diagnose and treat chronic diseases and the lack of skills to meet the new needs of patients could also prevent the healthcare system from handling such problems [41]. For example, in Mozambique, despite the adequacy and supply of insulin, the conditions of diabetic patients were not improved due to inadequate training of personnel and their lack of necessary skills [42]. In addition, the current personnel face problems with these issues in terms of motivation and job satisfaction. The prevention, care, and management of chronic conditions and NCDs put a heavy workload on the shoulders of these individuals. This occurs when a wide variety of programs issued by the MoHME and the medical sciences universities are announced more frequently without clear priority and coordination. Consequently, each program would require a series of particular actions and interventions, specialized activities and documentation, and statistical activities that would increase the workload of the workforce and, as a result, cause motivational problems. Mannava [43] examined detection of oral precancerous lesions and indicated that lack of incentives, specifically the lack of the delineation of targets, and the absence of official orders mandating detection of lesions caused motivational problems [43]. These findings are consistent with the results of studies from Bangladesh [44–47] and Vietnam [48].

Another category obtained from data was infrastructure, which consisted of inconsistency between programs and infrastructure and capacity of medical sciences universities, shortcomings in the HIM system, weaknesses in evaluations, and lack of a comprehensive evaluation system.

Of note, the implementation of all programs in any field requires a series of platforms and infrastructure that are needed as inputs to provide services. The findings of Rawal and those of our study indicate that there was no suitable infrastructure for implementing the program for the prevention and control of NCDs [47]. Several key challenges were identified, including inadequate logistics, supplies and medications, inefficient referral mechanisms, and unavailability of systematic recording and reporting systems. These findings are in line with the results of studies from Bangladesh [44–47],Vietnam [48], and Mumbai [49]. In the field of HIM, the designed system did not comply with the priorities of the PHC one, and the requirements for the use of the system were not available. There was also no integrated information system in practice, and the existing system could not provide the necessary information for decision-making. On the other hand, there was no assurance of the accuracy of statistics and outputs, and the personnel did not have the essential skills and capabilities to utilize them and even work with existing systems. Structural platforms and equipment for information and communication technology without increasing the ability of personnel to use them may impose high costs on the healthcare system and do not yield favorable outputs. Studies in developing countries have also indicated unavailability of information and data, insufficient investments in HIM systems, the inappropriateness of indicators related to chronic diseases, ambiguous data conversion and management methods, lack of information distribution and their use for comprehensive planning for such diseases as the main challenges of the healthcare systems [50, 51]. Studies have also found that upgrading standards through retraining, job assistance, and electronic health systems for rapid and easy retrieval of information could effectively advance the program for the prevention and control of NCDs [51]. Other studies have further shown that statistical systems have not evolved in line with information technologies, and they were still being traditionally employed to record, analyze, and report data [40].

Another category was administrative, executive, and legal problems, which included non-allocation of organizational positions and administrative charts for personnel involved in the program, inadequacy of salaries and benefits with the volume of program activities, and lack of transparency in job descriptions at different units and deputies in relation to the program.

Non-allocation of organizational positions and administrative charts for personnel involved in the program was a new finding in the study. Despite the achievements in the field of PHC, organizational structures and contexts have failed to fully fit the program for the prevention and control of such diseases. Factors such as the vagueness of financial resources for dealing with NCDs, insufficient credits, the lack of authority to pay the personnel from existing funds, and lack of transparency in obtaining recruitment licenses and organizational charts could thus play important roles in the non-allocation of organizational positions and administrative charts [52]. As mentioned in the human resource category, all these factors would reduce healthcare workers' motivation and job satisfaction. In some cases, their desire to work in the healthcare sector decrease, and they may ask to move to other units. Thus, it is necessary to review the organizational charts in accordance with the needs of the community and the action plan for the prevention and control of NCDs, and even give authority to local and regional managers to organize, review, and move the personnel in the charts with regard to supervision mechanisms from higher levels. One of the other challenges was the incompatibility of salaries and benefits with the tasks performed, which was especially of utmost importance among the permanent and contractual workforce. Lack of organizational positions and transparency in job classification and descriptions could further lead to paying improper salaries and benefits to some healthcare workers, which are significantly lower from their permanent counterparts. As a result, it discourages the personnel from improving their performance and providing high-quality, safe services. Our results in this concern are in concordance with the findings of Rawal [44].

According to the inter-sectoral collaboration category, the challenges consist of weaknesses in the understanding of program policies and objectives by units, organizations, and other bodies, weaknesses in the skills related to seeking the support of experts and managers in terms of attracting intersectoral collaboration and the lack of the transparency with respect to the role of NGOs, charities, and volunteer groups. Inter-sectoral collaboration on the program for the prevention and control of NCDs is the main subject described by Russell [29], Samb [27], Tuangratananon [12], Vali [13], and Mannava [43]. In this respect, the evaluation study by the Supreme Council of Health and Food Security found that a significant part of the inter-sectoral approvals had not been operationalized [33, 53]. A review of the approvals of the specialized working groups in the Supreme Council of Health and Food Security in Iran's provinces had also demonstrated that such approvals in the field of NCDs were negligible, and the weaknesses in inter-sectoral collaboration had been further mentioned [54, 55]. The lack of motivation in managers and experts due to weak and incomplete infrastructure and the lack of clear visions and comprehensive plans regarding the duties and roles of other bodies and institutions affiliated with healthcare had also induced the ambiguous expectations of the healthcare sector from other agencies. The other side of the issue for the lack of success was associated with the lack of appreciation for the cooperation of other organizations and agencies by the MoHME and the healthcare sector. In this way, the activities of partner organizations in advancing the goals of most programs were ignored, and success and progress were merely attributed to the MoHME and the medical sciences universities alone. In one study, the subtle point of the collaboration between the MoHME and other agencies had been further underscored. In this way, the MoHME could have a share in the social components affecting health, but the question was whether the given ministry was aware of its share and role and had a written plan for them or not. Moreover, the think win-win from the MoHME and other agencies and institutions could be a key element in advancing healthcare goals [56]. It was also necessary to determine the share of inter-sectoral collaboration in all programs issued by the MoHME, empower managers and experts of the healthcare system at all senior and executive levels to the lowest ones, monitor and evaluate joint programs, and provide accurate executive instructions and procedures. In the field of inter-sectoral collaboration, the capacities of NGOs, particularly charities and public participation, should be considered, but as indicated by participants, there were fundamental weaknesses in this regard. According to the findings of Gholamzadeh [57] charities could also have the potential capacity to provide such services in all three areas of prevention, treatment, and rehabilitation because these institutions have the deepest and closest relationships with the members of society, above all the deprived ones. The government could thus easily implement the capacity of charities to produce, disseminate, and increase public awareness regarding NCDs, conduct health-related research and use the results of such studies, finance healthcare services, and change social attitudes toward NCDs [51, 57].

Another challenge was concerned with the management and policy-making, including insufficient support of national and regional policy-makers and lack of understanding of the associated policies, individual-dependent programs, and weaknesses in evidence-based decision-making and policy-making system. Accordingly, improper expectations of some representatives and officials, political tendencies, lack of prioritization of PHC and advancements in the field of treatment, the declaration of many programs and plans without any coordination and prioritization, and as a result, the busy times of university presidents and deputies have reduced attention and supervision to the program for the prevention and control of NCDs [35, 40].

In the research by Amerzadeh political support and commitment were identified as factors affecting the prevention and control of NCDs [11]. Also, another issue presented as a challenge to implementing the program for the prevention and control of NCDs was the dependence of plans on individuals. Due to the lack of clear visions and comprehensive plans regarding the duties and roles of other bodies and institutions associated with the program for the prevention and control of NCDs, with the relocations of managers, especially those at the senior levels, other executive and expert levels could also be affected, and political support for programs might be even shrouded in series of ambiguities. Another problem was the weaknesses in evidence-based decisions. Factors such as the lack of comprehensive systems to the documentation of studies and evidence, absence of the understanding of the importance of policy-making, insufficient cognition about the programs and the current situation, unclear research priorities in the healthcare system, unawareness of policy-makers and planners regarding studies and evidence provided for this purpose in creating the given problem were also discussed [58].

## Conclusion

Unfortunately, the traditional system of PHC, which is mainly concentrated in rural areas, is incapable of providing complex, integrated, multi-sectoral, and continuous measures and interventions required for chronic conditions and NCDs. Such diseases demand a multifaceted response from the healthcare systems that must be accompanied by a structure of constant and sustainable healthcare, evidence-based interventions, effective public policies to address key risk factors, health professionals with diverse skills, appropriate technologies, reliable HIM systems, and available and secure health facilities in a stable and continuous manner. These interventions would be only possible with active healthcare systems that provide education and disease prevention services alongside integrated care and inter-sectoral collaboration beyond the healthcare sector.

Our results suggest that lack of financial resources were the most significant challenge interfering with the successful implementation of the program for the prevention and control of NCDs, but strengthening the healthcare system in the field of prevention of such diseases demanded more innovative measures and methods, such as coordinated efforts in various clinical, political, social areas with designated leadership, the use of feedback loops, the involvement of the public and physicians as partners for change, commitment to the requirements and goals of the program, and focus on the value of interdisciplinary and inter-organizational relationships with no need for financing.

### Limitation and strengths

This study's findings should be trusted despite some limitations. One of the main limitations was the widespread prevalence of COVID-19, which prevented some top managers from participating in the in-person interviews due to their busy schedules and adherence to health protocols. Therefore, the interviews were conducted via phone calls. Despite these limitations, our study is the first one to explore challenges facing implementing the program for the prevention and control of NCDs in Iran from the viewpoints of senior managers with a qualitative approach. It provides valuable information for health professionals, policy-makers, and government officials to empower the dimensions of governance, cope with some risk factors, and be responsive in an effective and timely manner to NCDs.

### Recommendations for future studies

It is suggested that future research explore challenges facing implementing the program for the prevention and control of NCDs in Iran from the middle level or operative managers' viewpoints. It is also suggested that quantitative studies be conducted to better understand other challenges and plan and take actions to improve them.

## Data Availability

The datasets used and/or analyzed during the current study are available from the corresponding author on reasonable request.

## Abbreviations

NCDs: Non-communicable Diseases
PHC: Primary Healthcare
HR: Human Resources
HIM: Health Information Management
NGOs: Non-governmental Organizations
DALY: Disability-Adjusted life Years
WHO: World Health Organization
MoHME: Ministry of Health and Medical Education
